# Assessing the cryptic invasion of a domestic conspecific: American mink in their native range

**DOI:** 10.1002/ece3.630

**Published:** 2013-06-11

**Authors:** Kaela B Beauclerc, Jeff Bowman, Albrecht I Schulte-Hostedde

**Affiliations:** 1 Wildlife Research and Development Section, Ontario Ministry of Natural ResourcesTrent University DNA Building, 2140 East Bank Drive, Peterborough, Ontario, K9J 7B8, Canada; 2 Department of Biology, Laurentian UniversityRamsay Lake Road, Sudbury, Ontario, P3E 2C6, Canada

**Keywords:** Domestic, hybridization, invasion, mink farm, *Neovison vison*, outbreeding depression

## Abstract

Control of invasions is facilitated by their early detection, but this may be difficult when invasions are cryptic due to similarity between invaders and native species. Domesticated conspecifics offer an interesting example of cryptic invasions because they have the ability to hybridize with their native counterparts, and can thus facilitate the introgression of maladaptive genes. We assessed the cryptic invasion of escaped domestic American mink (*Neovison vison*) within their native range. Feral mink are a known alien invader in many parts of the world, but invasion of their native range is not well understood. We genetically profiled 233 captive domestic mink from different farms in Ontario, Canada and 299 free-ranging mink from Ontario, and used assignments tests to ascertain genetic ancestries of free-ranging animals. We found that 18% of free-ranging mink were either escaped domestic animals or hybrids, and a tree regression showed that these domestic genotypes were most likely to occur south of a latitude of 43.13°N, within the distribution of mink farms in Ontario. Thus, domestic mink appear not to have established populations in Ontario in locations without fur farms. We suspect that maladaptation of domestic mink and outbreeding depression of hybrid and introgressed mink have limited their spread. Mink farm density and proximity to mink farms were not important predictors of domestic genotypes but rather, certain mink farms appeared to be important sources of escaped domestic animals. Our results show that not all mink farms are equal with respect to biosecurity, and thus that the spread of domestic genotypes can be mitigated by improved biosecurity.

## Introduction

The interaction between domesticated species and their wild relatives represents one of the many threats to wild populations and thus biodiversity. For example, many emerging infectious diseases in wildlife have reservoirs in farmed or domestic species, including chronic wasting disease and bovine tuberculosis (Williams et al. 2002; Woodroffe et al. 2009). The potential effects of domestic animals on wild populations can be further magnified if the domestic species become feral. Once established, feral populations may compete with local species for resources such as food and habitat. Australian dingoes (*Canis lupus dingo*) likely contributed in part to the extinction of the thylacine (*Thylacinus cynocephalus*) due to considerable overlap in prey size (Wroe et al. 2007). The presence of feral horses and donkeys reduces abundance, population growth, and the use of water sources by bighorn sheep, *Ovis canadensis* (Marshal et al. 2008; Ostermann-Kelm et al. 2008). The hybridization of feral populations with wild conspecifics is an additional effect that has numerous repercussions, including wasted reproductive effort, which may result from a lack of hybrid fertility (Rhymer and Simberloff 1996). For example, the embryos of European mink (*Mustela lutreola*) and feral American mink (*Neovison vison*) are not viable (Maran and Henttonen 1995). Of greater concern, however, is the introgression of maladaptive genes into the wild population; indeed, hybridization with feral populations has been shown to be quite extensive in some wild species (Beaumont et al. 2001; Norén et al. 2009; Godinho et al. 2011). Introgression can reduce fitness by introducing maladaptive genes and causing outbreeding depression through the loss of local adaptations and the breakdown of coadapted gene complexes (Rhymer and Simberloff 1996). For example, following several generations of hybridization between farmed and wild individuals, both rainbow trout (*Oncorhynchus mykiss*; Tymchuk et al. 2007) and Atlantic salmon (*Salmo salar*; McGinnity et al. 2003) had lower fitness than their purely wild counterparts.

The demographic impact of feral domestic individuals on the broader wild population largely depends on the feral animal's ability to survive and disperse. Species with broad ecological tolerances are often more successful at invading novel environments relative to those that occupy specialized niches (Marvier et al. 2004). For example, an invasive benthic tunicate was found to be more thermotolerant and inhabited broader temperature ranges than the native tunicate species (Zerebecki and Sorte 2011), whereas the Chinese water deer (*Hydropotes inermis*) introduced to Great Britain has not become widely established, as it appears to be restricted by habitat requirements (Ward 2005). Genetic diversity is also an important component to a species' invasiveness. Several invasive plants have been found to possess greater genetic diversity than their source populations, including recombinant genotypes due to hybridization between individuals from multiple source populations, that may facilitate adaptive potential during invasion (Lavergne and Molofsky 2007; Prentis et al. 2008; Le Roux et al. 2011). In contrast, however, many successfully introduced species were initially founded by only a small number of individuals and show reduced genetic variation relative to their native populations. For example, only six moose (*Alces alces*) were introduced to Newfoundland, Canada, but the population now numbers >150,000 despite a loss of 22–46% of genetic variation (Broders et al. 1999). Perhaps at its simplest, the release of large numbers of individuals and frequent release events (high propagule pressure) increases the likelihood that a species will become established outside its native range, often irrespective of life history or environmental conditions (Lockwood et al. 2005).

**Figure 1:**
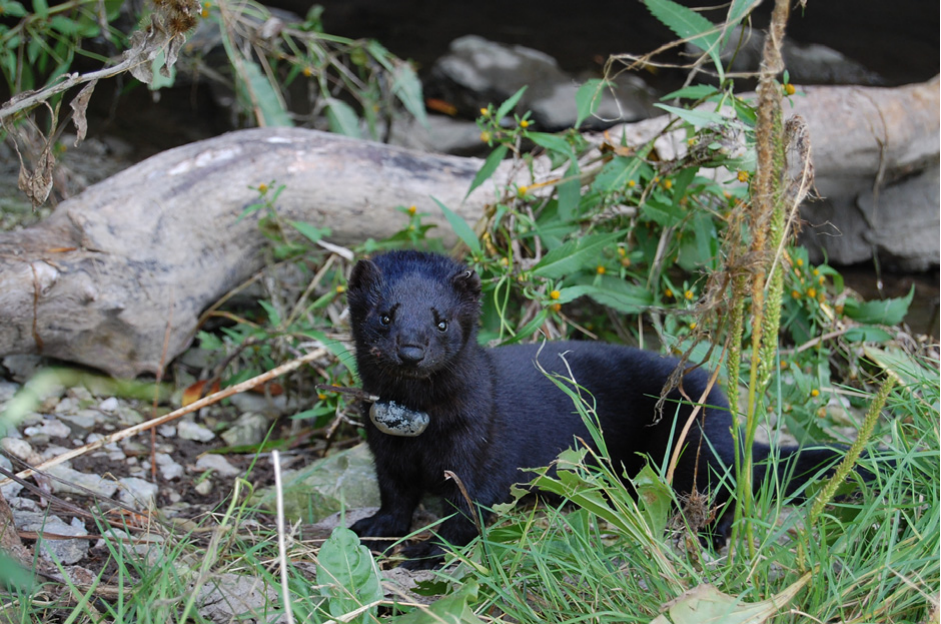
An American mink (*Neovison vison*) with a hybrid domestic–wild genotype photographed on the shore of the Niagara River in Ontario, Canada (Photo taken by Larissa Nituch).

The American mink is a semiaquatic mustelid endemic to North America that has been trapped for its fur for centuries (Fig. 1; Joergensen 1985). Since the late 1800s, mink have been domesticated and raised on fur farms, both within and outside of their native range. One of the consequences of domestication and husbandry of mink on farms has been the escape of these mink into the natural environment via accidental and intentional releases. The establishment of feral mink populations and the concomitant ecological effects have been documented extensively in Europe and South America, outside of the species native range (Macdonald and Harrington 2003; Bonesi and Palazon 2007). It is not surprising that a mink invasion in these jurisdictions would be easy to detect, because there is no native conspecific to confuse observers. More recently, however, Bowman et al. (2007) showed that domestic mink are also escaping within their native range in North America. Although the invasive nature of American mink in its nonnative range has been appreciated (Bonesi and Palazon 2007; Bryce et al. 2011), the potential for invasion has not been widely recognized in the native range of mink, perhaps due to the cryptic nature of the invasion (Bowman et al. 2007). Although domestic mink are genetically and phenotypically distinct from wild American mink (Kidd et al. 2009; Bowman et al. 2012), the distinctions between these groups are subtle. Furthermore, the introgression of genes from domestic to wild populations in Canada has been documented (Kidd et al. 2009), and as such, the footprint of a domestic mink invasion may be especially pervasive and difficult to detect due to the presence of introgressed individuals.

As has been demonstrated in Europe (Bonesi and Palazon 2007), mink farms can have frequent escape events, leading to the high propagule pressure that facilitates invasion (Lockwood et al. 2005; Zalewski et al. 2011), including establishment of feral mink in regions without mink farms (Bonesi and Palazon 2007). Furthermore, feral mink outside of North America often appear to exhibit high fitness (Macdonald and Harrington 2003), and this has led frequently to high spread rates (1.5–20 km/year; Usher 1986; Ruiz-Olmo et al. 1997; Iordan et al. 2012), and a cascade of ecological damage (Macdonald and Harrington 2003; Bonesi and Palazon 2007). These are typical characteristics of an invasive species, and have led many jurisdictions with feral mink to invest heavily in control and eradication programs in attempts to remove mink and mitigate the ecological damage (Bonesi and Palazon 2007; Zuberogoitia et al. 2010; Iordan et al. 2012). Because of the cryptic nature of free-ranging domestic mink and their genes in North America, it is relatively unclear whether feral mink have become established in their native range, and whether the footprint of the invasion is of a similar spatial extent as in Europe, and other nonnative contexts. Identifying the spatial extent and ecological magnitude of a cryptic invasion by domestic mink in North America represents an important first step in assessing whether eradication and control plans are necessary, and in developing these plans. If feral mink in North America have high fitness then we expected to find evidence of the high spread rates typical in mink invasions from their nonnative range, and an overall extensive footprint of feral mink. This would include finding domestic mink and their alleles to be prevalent in the free-ranging mink population including in areas without mink farms. We tested these predictions by investigating the spatial distribution of free-ranging domestic and hybrid mink, relative to wild mink and mink farms, in Ontario, Canada.

## Materials and Methods

### Study area

Our Ontario study area consisted of highly fragmented Carolinian forest in the southwest, Great Lakes-St. Lawrence temperate-boreal ecotonal forest in the central part of the area, and boreal forest in the north (Rowe 1972). The south of the study area was a highly developed agriculture and urban landscape, whereas the north was heavily forested, and managed for timber and wood pulp. There are many lakes and waterways throughout the province, and historically, mink have occurred throughout the area. Trapping wild mink for fur occurred throughout the province.

**Figure 2:**
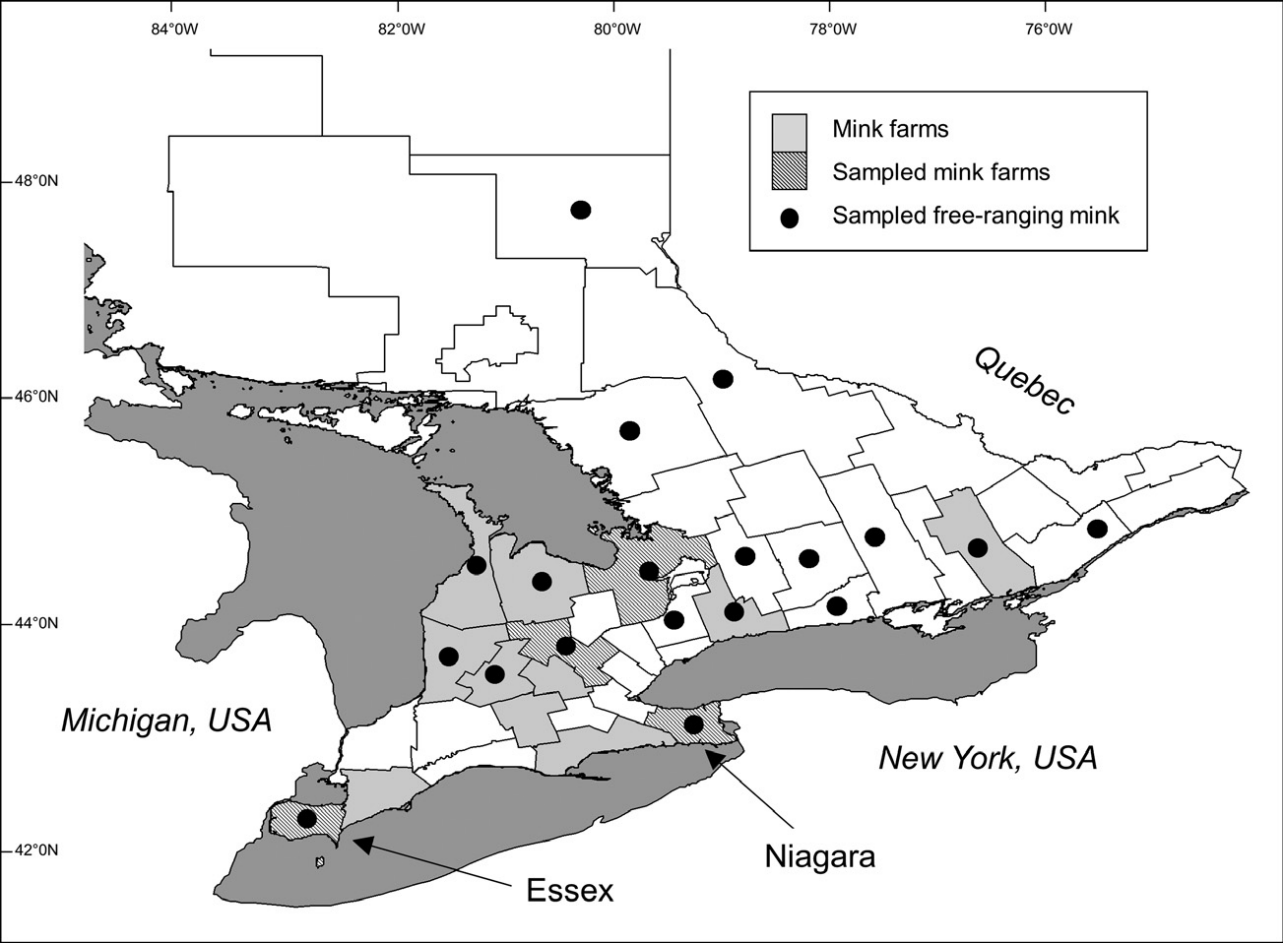
Location of study in Ontario, Canada where American mink (*Neovison vison*) were sampled. Mink were either captive domestic individuals (cross-hatched counties) or free-ranging domestic, hybrid, or wild individuals (black points). Gray shading indicates counties where mink farms occurred but were not sampled.

We stratified our mink sampling across Ontario's census counties, because this was the spatial scale for which we had complete data on the distribution of fur farms (Statistics Canada 2006). There were 60 mink farms reported in Ontario during the 2006 Census of Agriculture (Statistics Canada 2006), and about 350,000 domestic mink in those farms. The number of mink farms per county in Ontario varied from 0 to 11. There was a significant positive correlation between the number of mink farms per county and the number of individual domestic mink per county (*r* = 0.93, *P* < 0.001). Generally, most mink farms in Ontario were in the south of the province, and in particular, in the southwest. We sampled across the distribution of mink farms from the far southwest to the boreal forest in the north, where there were no mink farms (Fig. 2).

### Sample collection

Tissue samples were collected from 242 domestic and 321 free-ranging mink throughout the study area between 2005 and 2010 (Fig. 2). A subset of these were previously described and analyzed by Kidd et al. (2009); however, these individuals were profiled at additional microsatellite loci for the current study, as described below. Domestic mink consisted of five color phases (the genetically dominant “standard brown,” the lighter brown “pastel,” “black,” the black–brown hybrid “mahogany,” and the silver “iris”). Domestic mink originated from four different farms in Ontario, including: a Wellington County farm (*n* = 30 black mink); a Simcoe County farm (*n* = 29 mink with one brown individual and the remainder either black or black–brown hybrids); a Niagara farm (*n* = 20 iris and 20 mahogany mink); and an Essex County farm (*n* = 29 black, 28 brown, and 15 pastel) (Fig. 2). Mink from the latter two farms were the same individuals studied by Kidd et al. (2009). Carcasses were obtained from all farms during the pelting process, and muscle was taken during necropsy for genetic analysis.

Free-ranging mink were sampled from 19 counties throughout Ontario (Table 1, Fig. 2), and were obtained via several methods: targeted live trapping, collection by fur harvesters, and as road kills. Tissue samples from live-trapped individuals consisted of plucked hairs including the roots, or blood obtained by nicking the quick of a nail and stored on Whatman FTA cards. All livetrapped animals were captured and sampled in accordance with approved animal care protocols from Trent and Laurentian Universities. Carcasses from fur harvesters and road kills were necropsied, and muscle or spleen taken for genetic analysis.

**Table 1:** Free-ranging American mink (*Neovison vison*) samples successfully genotyped in a study of mink genetics in Ontario, Canada

County	*N*
Bruce	7
Durham	5
Essex	24
Frontenac	1
Grey	28
Hastings	6
Huron	9
Lanark	1
Leeds and Grenville	25
Niagara	69
Nipissing	1
Northumberland	3
Parry Sound	2
Perth	5
Peterborough	13
Simcoe	12
Timiskaming	56
Wellington	5
Victoria	7
York	20
Total	299

The number of individuals (*N*) sampled per county is shown.

Additionally, 58 historic specimens from several museums were sampled for genetic analysis. Tissue samples consisted of either small snips of skin and hair from the ventral suture of prepared skins, or scrapings of tissue adhering to skeletal elements. Individuals collected prior to the 1960s were targeted, as this is the time at which mink farms became abundant in Canada. Specimens ranged in date of origin from 1897 to 1974, with the majority (97%) from 1950 or earlier. Although the focus of our study was Ontario, specimens from Quebec (*n* = 21 of 58 samples) were also included given the limited number of historic samples available. Furthermore, no information existed as to the genetic population structure of mink in Canada; as such, we considered it reasonable to include adjacent geopolitical regions to obtain insight into the historical genetic constitution of Ontario mink.

### Microsatellite genotyping

Approximately 10 mg of tissue were dissolved in 500 μL of 1× lysis buffer (2 mol/L urea, 0.1 mol/L NaCl, 0.25% *n*-lauroyl sarcosine, 5 mmol/L 1,2 cyclohexanedinitrilotetraacetic acid (CDTA), and 0.05 mol/L Tris-HCl pH 8) and digested with 50 μL of proteinase K (20 mg/mL). Whole genomic DNA was extracted with the DNeasy Tissue Kit (Qiagen Inc., Valencia, CA) or an automated magnetic bead procedure using MagneSil PMPs (Promega, Madison, WI), and quantified with a NanoDrop 8000 (Thermo Fisher Scientific, Waltham, MA).

All samples were amplified at 16 microsatellite loci (Table 2), 10 of which were also used by Kidd et al. (2009). Forward or reverse primers were labeled with 6-FAM, HEX, or NED (Applied Biosystems, Foster City, CA). Polymerase chain reaction (PCR) amplifications were carried out in 10 μL reactions containing 5 ng DNA, 1× PCR buffer, 1.5 mmol/L MgCl_2_, 0.2 mmol/L dNTPs, primer concentrations as per Table 2, 0.2 μg/mL bovine serum albumin (BSA), and 0.5 U of *Taq* polymerase (BioShop Canada Inc., Burlington, ON, Canada). Thermal cycling used an Eppendorf Mastercycler Pro thermal cycler with an initial denaturation at 94°C for 5 min, followed by 30 cycles of denaturation at 94°C for 30 sec, annealing at the optimal *T*_A_ (see Table 2) for 1 min, and extension at 72°C for 1 min, and a final extension of 60°C for 45 min. Loci were optimized into eight reactions and pooled into three groups with 500 ROX for genotyping on an ABI 3730 automated sequencer. Fragment sizes were scored using Genemapper 4.0 (Applied Biosystems).

**Table 2:** Microsatellite loci and PCR conditions used to genotype American mink (*Neovison vison*) in a study of mink sampled in Ontario, Canada

PCR reaction	Locus^1^	Primer (μmol/L)	*T*_A_	Pooling volume (μL)	Size	*N*_A_	*H*_E_	*H*_O_	Source
Multiplex 1									
1	Mvi111 H	0.2	55	2	84–108	11	0.66	0.75	O'Connell et al. (1996)
2	Mvi1006 F	0.5	60	4	136–168	14	0.64	0.81	Farid et al. (2004)
	Mvi1272 H	0.4	60		163–183	11	0.74	0.79	Vincent et al. (2003)
3	Mvi099 F	0.5	59	2	320–362	20	0.74	0.84	Fleming et al. (1999)
	Mvi4001 N	0.2	59		223–233	6	0.47	0.49	Anistoroaei et al. (2006)
4	Mvi114 N	0.3	61	4	62–82	10	0.74	0.83	O'Connell et al. (1996)
	Mvi1302 H	0.6	61		203–223	11	0.65	0.80	Vincent et al. (2003)
Multiplex 2									
5	Mvi1016 F	0.4	63	N/A	216–236	11	0.75	0.82	Farid et al. (2004)
	Mvi1321 F	0.1	63	N/A	90–116	11	0.69	0.80	Vincent et al. (2003)
	Mvi2243 N	0.2	63	N/A	123–157	13	0.64	0.71	Vincent et al. (2003)
Multiplex 3									
6	Mvi1354 F	0.4	60	2	174–208	15	0.69	0.82	Vincent et al. (2003)
	Mvi002 N	0.4	60		176–188	6	0.09	0.11	Fleming et al. (1999)
	Mvi072 F	0.2	60		257–273	9	0.55	0.65	Fleming et al. (1999)
7	Mvi087 H	0.2	60	4	70–84	6	0.21	0.60	O'Connell et al. (1996)
	Mvi1342 H	0.6	60		138–174	14	0.66	0.81	Vincent et al. (2003)
8	Mvi075 F	0.2	60	1	105–139	14	0.81	0.86	Fleming et al. (1999)

*T*_A_ is annealing temperature. Also shown are the number of alleles (*N*_A_), expected heterozygosity (*H*_E_), and observed heterozygosity (*H*_O_) calculated using contemporary domestic and free-ranging individuals.

1 H, F, and N refer to HEX, 6-FAM, and NED fluorescent labels, respectively.

### Historic samples

Historic samples were extracted in a laboratory dedicated to processing ancient DNA at Trent University in Peterborough, Ontario. Separate rooms exist for handling reagents and tissue/DNA, and no mustelid species had previously been processed in these rooms. Fresh reagents and supplies were used at all stages. Samples were extracted with the Qiagen DNeasy Tissue kit (Qiagen Inc.) according to the manufacturer's instructions, with blanks incorporated for every 10 samples. PCR was carried out with the same conditions as the contemporary samples, except 50 cycles were used for the denaturing, annealing, and extension steps.

### Genetic diversity

Microsatellite diversity indices (number and frequencies of alleles, observed and expected heterozygosity) were calculated using Genalex v. 6.3 (Peakall and Smouse 2006). Conformation to Hardy–Weinberg equilibrium (HWE) and pairwise linkage disequilibrium (LD) were evaluated in Genepop v.4.0.10 (Rousset 2008) by estimation of exact *P*-values and the log likelihood ratio statistic, respectively, using a Markov Chain Monte Carlo (MCMC) method with 5000 dememorization steps, 1000 batches, and 5000 iterations per batch. HWE and LD were tested globally as well as for each separate domestic group and free-ranging site for which *N* > 5. Both HWE and LD were adjusted for multiple comparisons using Bonferroni's correction. Allelic richness was assessed using rarefaction to standardize groups to the smallest sample size using HP-Rare (Kalinoski 2005). Separate values were calculated for each locus using the contemporary domestic and free-ranging samples, for the historic samples, and for the genetic clusters identified through assignment analyses with *K* = 2 (see below).

### Population structure

Genetic structure and ancestry of American mink in Ontario were evaluated using Bayesian assignment tests as implemented in Structure v.2.2 (Pritchard et al. 2000). We analyzed the complete contemporary dataset (domestic and free-ranging samples, but no historic samples; *n* = 532) with the admixture ancestry model and correlated allele frequencies; default settings were used for all other parameters. The MCMC chain used a burn-in of 500,000 and 10^6^ iterations. Five simulations were run for each assumed number of clusters (*K*) ranging from *K* = 1 to *K* = 10. The average log likelihood of *K* from the five replicates was used to infer the most likely number of clusters according to the method of Evanno et al. (2005). Cluster membership for each individual was evaluated using the average ancestry coefficient (*q*) from the five replicates. An individual was assigned to a single cluster if it had a *q* > 0.8, whereas it was assigned jointly to two or more clusters such that the minimum sum of the largest *q* values was >0.8. Simulations have previously shown the criterion of *q* > 0.8 to be of sufficient power to correctly assign 96% of mink to either domestic, wild, or hybrid groups (Kidd et al. 2009). A second assignment analysis was conducted with Structure for the historic samples in combination with the contemporary free-ranging and domestic samples, using the same parameters at *K* = 2 and *K* = 7.

We did not want to attribute population clustering to mink if the underlying process was actually a gradient of isolation-by-distance (Schwartz and McKelvey 2009). Therefore, we used a Mantel test to assess isolation-by-distance for all wild mink genotypes (i.e., we did not include free-ranging domestic or hybrid genotypes). We either used precise GPS locations of capture sites for each mink to represent spatial coordinates, or the geographic centroid of the township of capture, where no GPS location was available. We used the individual-based genetic distance matrix introduced by Smouse and Peakall (1999) and implemented in Genalex v. 6.3 (Peakall and Smouse 2006) to conduct the Mantel test.

### Spatial analysis

In order to explore the factors important to determining the presence of farm escapees in Ontario, we used a regression tree to identify the variables that predicted the probability of being classified as a farm escapee (e.g., Thomassen et al. 2010). Regression trees use a binary recursive partitioning approach to split data, including spatial location data, into homogenous clusters. Our response variable, the probability of being a domestic mink, was the *q* value obtained from Structure for *K* = 2. For predictor variables, we included the latitude and longitude for the site of capture (as a measure of location and a proxy for spatial pattern), the density of farms within the township of capture, and the distance from site of capture to the nearest farm. As with the Mantel test, we either used precise GPS locations of capture sites, or the geographic centroid of the township of capture, where no GPS location was available. The distance to the nearest farm was calculated in ArcGIS 9.2 (ESRI, Redlands, CA). The number of farms per township was obtained from Statistics Canada (2006). Farm density was calculated for each county as the number of farms per km^2^. The regression tree was set to include a minimum node size of 30, and tree analyses were conducted in R (R Core Development Team 2011).

## Results

### Genetic diversity

In total, 233 domestic individuals and 299 free-ranging individuals (94.5% of 563 mink) were successfully genotyped at 10 or more loci and used for analyses. The number of alleles per locus ranged from six to 20, while observed and expected heterozygosity ranged from 0.11 to 0.84 and 0.09 to 0.81, respectively (Table 2). Initial analyses showed an extreme heterozygote deficiency for locus Mvi087, which likely reflected the presence of null alleles; as such, this locus was removed from further analyses. Globally, all loci deviated significantly from HWE due to a deficiency of heterozygotes (*P* < 0.01). When separated into groups (color phase for domestic, sites for free-ranging), two free-ranging (Niagara and Timiskaming) and three domestic (brown, iris, mahogany) groups continued to deviate from HWE at Bonferroni-corrected *P* < 0.05. Twenty-seven pairs of loci deviated from LD at Bonferroni-corrected *P* < 0.05 when all samples were considered globally; however, when subdivided into groups, only loci Mvi1354-Mvi072 continued to deviate from LD at the Niagara site.

### Population structure

Bayesian analysis identified *K* = 2 as the most likely number of clusters for the data, and similar to the finding of Kidd et al. (2009), *K* = 2 differentiated between wild and domestic mink genotypes. Overall, 82% of free-ranging mink belonged to the wild type (*n* = 245), 13% were identified as farm escapees (*n* = 39), and 5% were domestic–wild hybrids (*n* = 15). The mean (SE) membership of free-ranging individuals in the wild cluster was 0.82 (0.02). The majority of feral and hybrid mink (49 of 54 individuals) occurred in two locations, Niagara and Essex, which had been previously studied by Kidd et al. (2009). The remaining feral and hybrid individuals included two feral mink sampled in Huron and a single hybrid in each of Grey, Leeds and Grenville, and Simcoe counties. Expected heterozygosity did not differ among domestic, hybrid, and wild mink (Table 3; Friedman's test, *P* = 0.69); however, allelic richness was significantly different among the groups (Friedman's test, *P* = 0.02). A post hoc Dunn's test showed that this was due to lower allelic richness in the domestic group compared to the wild group (*P* = 0.003).

**Table 3:** Expected heterozygosity (*H*_E_) and allelic richness (*A*_R_) for wild, domestic, and hybrid American mink (*Neovison vison*) sampled in Ontario, Canada

Locus	*H*_E_				*A*_R_			
	
	Wild	Domestic	Hybrid	Historic	Wild	Domestic	Hybrid	Historic
Mvi1006	0.85	0.76	0.80	0.88	7.81	7.27	6.09	10.04
Mvi111	0.69	0.75	0.81	0.63	6.29	7.17	5.08	6.42
Mvi1272	0.76	0.80	0.68	0.77	6.19	3.99	6.25	5.59
Mvi1302	0.79	0.71	0.82	0.78	6.04	6.74	5.69	6.08
Mvi114	0.85	0.75	0.85	0.80	7.34	7.68	5.77	6.51
Mvi4001	0.38	0.60	0.52	0.50	3.84	3.67	3.49	4.49
Mvi099	0.78	0.84	0.80	0.81	6.91	6.40	7.48	8.38
Mvi1321	0.78	0.80	0.78	0.81	6.57	6.59	6.55	6.50
Mvi1016	0.80	0.81	0.80	0.71	6.71	6.91	6.64	6.50
Mvi2243	0.67	0.74	0.71	0.69	4.72	4.00	5.81	4.10
Mvi075	0.87	0.81	0.86	0.84	8.49	9.23	6.85	8.55
Mvi1354	0.85	0.64	0.84	0.79	7.62	8.14	4.39	7.12
Mvi072	0.73	0.53	0.56	0.54	5.25	4.67	4.02	3.82
Mvi1342	0.79	0.78	0.80	0.80	6.57	6.00	5.96	6.27
Mvi002	0.15	0.07	0.18	0.26	2.32	2.67	1.68	2.54
Mean (SE)	0.72 (0.05)	0.69 (0.05)	0.72 (0.05)	0.71 (0.04)	6.18 (0.41)	6.08 (0.48)	5.45 (0.39)	6.19 (0.50)

Free-ranging mink are included in the group to which they were genetically assigned, and all historic samples are shown as a separate group. Allelic richness was adjusted for sample size using rarefaction.

**Figure 3:**
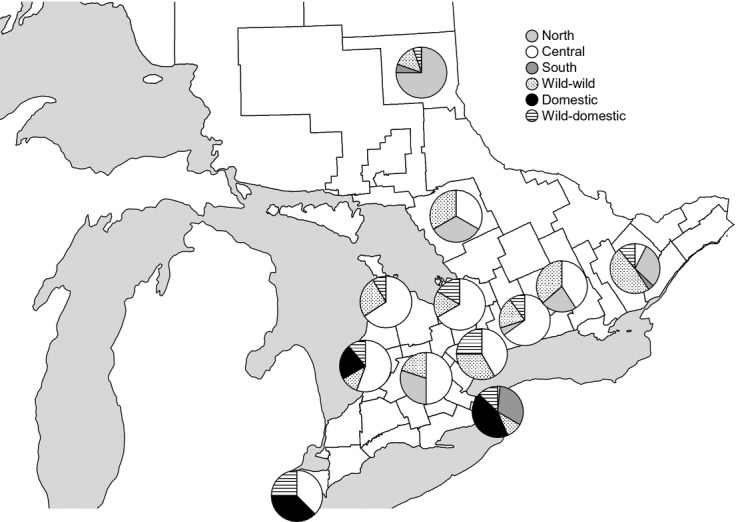
Distribution of clusters of free-ranging mink (*Neovison vison*) from Ontario identified using Structure with *K* = 7. To increase clarity, most sample sites with *N* < 10 were consolidated with adjacent sites into single charts. Three wild clusters are shown, and the four domestic color lines are collapsed into a single, fourth group. Two additional groups are depicted: domestic–wild hybrids and wild–wild hybrids. The wild–wild group consists of individuals not assigned to any single wild cluster with *q* > 0.8, but for which total *q*_wild_ > 0.8.

The same Structure analysis demonstrated a second peak at *K* = 7 (Δ*K* = 100.11) that identified three wild and four domestic clusters. The domestic clusters largely corresponded to the iris, black, mahogany, and brown (including standard brown and pastel) color phases. For all except mahogany, >70% of individuals were assigned to a single cluster at *q* > 0.8; remaining individuals were largely hybrids of two or more domestic clusters. Only 58% of mahogany individuals belonged to the mahogany cluster, while 26% were domestic–domestic hybrids (predominantly composed of the black and mahogany clusters). The three wild clusters were largely structured geographically across Ontario; as such, we refer to them as “South,” “North,” and “Central” (Fig. 3). All individuals belonging to the Central cluster were found south of Lake Huron, although several geographically southern sites contained individuals that were assigned to the North cluster (Fig. 3). Seventy percent of individuals classified as North were found in a single site, Timiskaming, which also had the largest number of private alleles (11). The third wild cluster (South) was dominated by individuals from Niagara, and indeed, most (73%) free-ranging Niagara samples classified as wild belonged to this cluster. Notably, however, three individuals from the northern site of Timiskaming were also assigned to this cluster (Fig. 3). Overall, 26% of wild free-ranging individuals were hybrids of the wild clusters.

When we considered seven clusters, the number of free-ranging individuals assigned as either domestic or domestic–wild hybrids was altered slightly compared to *K* = 2. The overall number of domestic escapees decreased from 39 to 35, while the number of domestic–wild hybrids increased from 15 to 32; the number of domestic or hybrid mink increased from 54 to 67, and the number of sites at which they were found also increased from 5 to 9. However, many of the additional hybrid individuals were predominantly assigned to the wild clusters, with wild membership >0.7.

After removing all hybrid and domestic individuals from the free-ranging mink samples, we observed no evidence of isolation-by-distance among the remaining wild mink genotypes (*r* = 0.028, *n* = 245, *P* = 0.215).

**Figure 4:**
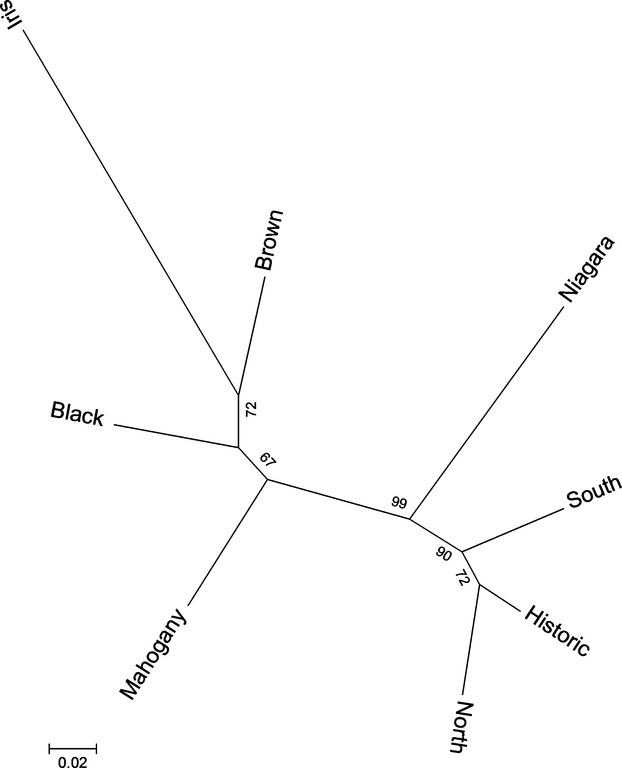
Neighbor-joining tree for genetic distance (*D*_A_) between wild and domestic clusters of American mink (*Neovison vison*) identified using Structure with *K* = 7. The historic samples are also shown. Significance was assessed using 1000 bootstrap replications. *[Correction added on 14 June 2013, after first online publication: The labels ‘South’ and ‘Central’ have been corrected from ‘Niagara’ and ‘South’, respectively.]

Given the high proportion of domestic escapees and hybrids found at the Niagara site, we questioned whether the South cluster was indeed a wild cluster, or another domestic cluster for which we had not sampled any known individuals. Closer examination of the individuals belonging to this cluster showed that, at *K* = 2, all but one were assigned to the wild cluster (the remaining individual being a domestic–wild hybrid). To further investigate the ancestry of this group, we used the analysis of molecular variance framework in Genalex to calculate population pairwise *F*_ST_ values for each of the seven clusters after removing all hybrids (both wild–wild and domestic–wild). Although all values were significant based on 9999 permutations, pairwise *F*_ST_ between the South cluster and other wild clusters was lower than *F*_ST_ between South and any of the domestic clusters (Table 4). We also constructed a phylogenetic tree in Poptree 2 (Takezaki et al. 2010) using the genetic distance (*D*_A_) between clusters and the neighbor-joining algorithm, as recommended by Takezaki and Nei (2008). Significance was assessed using 1000 bootstrap replications. Once again, this analysis showed that the South cluster was more similar to the other wild clusters than to the domestic clusters (Fig. 4). These analyses also demonstrated the divergence of the iris color phase from the remaining domestic lines, as was found by Kidd et al. (2009).

**Table 4:** Pairwise *F*_ST_ values for wild (italics) and domestic clusters of American mink (*Neovison vison*) identified using a Bayesian assignment test

	*Central*	*North*	*South*	Iris	Black	Mahogany
*North*	0.051					
*South*	0.077	0.087				
Iris	0.176	0.176	0.189			
Black	0.120	0.111	0.121	0.135		
Mahogany	0.102	0.089	0.101	0.130	0.084	
Brown	0.117	0.087	0.118	0.135	0.070	0.092

Mink were all sampled in Ontario, Canada, either on mink farms or as free-ranging individuals. All values were significant using 9999 permutations.

### Spatial analyses

**Figure 5:**
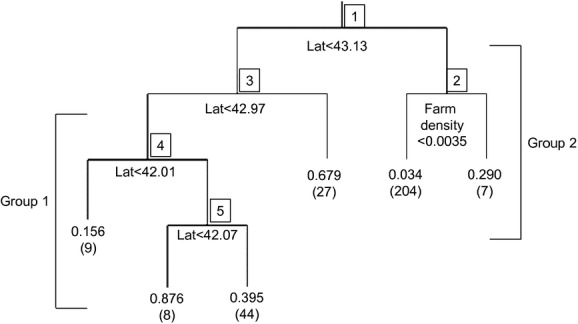
Regression tree of the probability of being a domestic mink for free-ranging American mink (*Neovison vison*) sampled in Ontario, Canada. Node and group numbers are to clarify discussion in the text only. Values at branch tips are the probability of being domestic (as determined in genetic assignment program Structure using *K* = 2 groups) and number of samples (in parentheses). Latitude is decimal degrees and farm density is mink farms/km^2^.

The primary split (node 1) in the regression tree identified latitude <43.13°N as the best predictor of a free-ranging individual's probability of being classified as domestic (Fig. 5). This latitude separated nearly all individuals in Essex and Niagara (hereafter called Group 1; *n* = 88) which had a 0.502 probability (*P*) of being domestic, from remaining sites (Group 2; *n* = 211). Although Group 2 individuals had an overall low probability of being domestic (0.043), those in sites with a farm density of >0.0035 per km^2^ (node 2) were more likely to be domestic (*P* = 0.290; *n* = 7) compared to those in low farm-density sites (*P* = 0.034; *n* = 204). Group 1 individuals were further subdivided based on latitude, rather than the farm density or proximity to farm predictors. The first split (node 3) identified 27 individuals from north of latitude 42.97°N (but still south of 43.13°N) as having a high probability of being domestic (0.679). These 27 individuals were from Niagara, primarily from a single township with a relatively high density of farms (0.0048 per km^2^), and they were captured an average of 4.29 km away from a known farm (range = 0.1–19.13 km; median = 2.38 km). Node 4 separated nine individuals from south of latitude 42.01°N (in Essex county) that had a low probability of being domestic (0.156), whereas node 5 identified individuals from Essex that had a very high probability of being domestic (0.876). Although both of these clusters of Essex individuals were captured in the same county with a relatively high farm density (0.0072 per km^2^), the two groups were captured 9.92–16.59 km and 2.3–4.42 km away from a known farm, respectively. The split at node 5 also included the remaining individuals from Essex and Niagara (*n* = 44), which had a lower probability of being domestic (0.395). Most (75%) individuals belonging to this group were from three sites in Niagara that had no known farms.

### Historic samples

Profiles comprised of 10 or more loci were obtained for 21 historic samples (36% of 58 individuals; Table 5). Employing Structure with *K* = 2 (and also including all contemporary samples previously discussed), mean (SE) membership of historic individuals in the wild cluster was 0.93 (0.03). For *K* = 7, mean membership was greatest for the North cluster (0.49), followed by the Central cluster (0.30). When individuals were assigned to the cluster for which they had the greatest membership, 11 individuals were assigned to the North cluster, seven to the Central, and three to the South cluster. The neighbor-joining tree (Fig. 4) also demonstrated this close affiliation of the historic samples with the North cluster. Three new alleles were identified in two individuals: one for locus Mvi099, found in an individual collected in 1918 from Preston, Ontario, and two for locus Mvi1006 in a single individual collected in 1900 from Algonquin Park, Ontario. The historic samples did not differ significantly in expected heterozygosity or allelic richness (Friedman's test, *P* > 0.05) from the contemporary wild, hybrid, or domestic groups (Table 3).

**Table 5:** Historic museum specimens of free-ranging American mink (*Neovison vison*) sampled for a study of mink population genetics in Ontario, Canada

Accession No.	Sex	Specimen	Province	Location	Year
CMNA 1691	M	Complete skeleton	ON	Peel Region; Lorne Park	
CMNA 3482	M	Complete skeleton	ON	Preston	1918
CMNA 36400	M	Study skin, skeleton, bones	ON	Ottawa, Dunbar Bridge	1960
CMNA 6759	F	Study skin, complete skeleton	ON	Rockwood	1916
CMNA 685	M	Study skin	ON	Algonquin Park	1900
CMNA 18970	M	Study skin, complete skeleton	QC	Area NW of Laurentides Park	1946
CMNA 18971	F	Study skin, complete skeleton	QC	Area NW of Laurentides Park	1946
CMNA 19370	M	Study skin, complete skeleton	QC	Lake Albanel	1947
CMNA 19371	M	Study skin, complete skeleton	QC	Lake Albanel	1947
CMNA 20429	M	Study skin, complete skeleton	QC	Saguenay-St. Lawrence	1950
CMNA 5019	–	Complete skeleton	QC	Pontiac Regional County	1923
CMNA 5280	–	Complete skeleton	QC	Pontiac; Spruce Grove	1924
CMNA 8736	–	Complete skeleton	QC	Pontiac; Eureka Lake	1927
CMNA 8737	–	Complete skeleton	QC	Pontiac; Eureka Lake	1927
CMNA 8739	–	Complete skeleton	QC	Pontiac; Eureka Lake	1927
FMNH 199825	–	Skull	ON	Quetico	1931
NBM B.CA.4	F	–	ON	Lanark county	1974
NBM S.C.G. 4	–	–	ON	–	1963
ROM 32.4.13.1	F	Skin, skull	ON	Muskoka Co	1932
ROM 33.9.30.4	M	Skin, skull	ON	Manitoulin Co.	1933
ROM 35.9.14.15	F	Skin, skull	ON	Nipissing Co.	1935

Specimens were sampled in either the Canadian province of Ontario (ON) or Quebec (QC). CMNA, Canadian Museum of Nature; FMNH, Field Museum of Natural History; NBM, New Brunswick Museum; ROM, Royal Ontario Museum.

## Discussion

Domestic mink appear to not have spread in Ontario, within the native range of American mink, as extensively as they have done in many places outside of the mink's native range. Escaped domestic mink and their genes were relatively rare across most of the study area, and the footprint of escaped mink was closely associated with mink farms. We detected no domestic mink beyond the distribution of mink farms in Ontario. These findings lead us to suspect that unlike in some nonnative contexts (Macdonald and Harrington 2003), escaped domestic mink in North America may have low fitness.

Domestic American mink were introduced to Europe as early as the 1920s, and today feral mink occur throughout Europe, with established populations in the United Kingdom, Fennoscandia, parts of France and Spain, and many eastern European countries (Bonesi and Palazon 2007, Zalewski et al. 2011). The established populations in Europe include many jurisdictions without current fur farms. In fact, Hammershøj et al. (2006) suggested that the establishment of feral mink populations in Europe may be enhanced in areas without farms due to reduced genetic swamping by recent domestic escapees and thus increased opportunities for natural selection. In contrast to the extensive spread of escaped domestic mink across Europe, the overall impact of domestic individuals in Ontario appeared to be largely restricted to a region of high mink farm density in the southwest of the province. Two counties in particular, Niagara and Essex, were highly mixed, accounting for 95% and 80% of all escapees and hybrids, respectively. This finding was supported by the tree regression, which identified mink sampled south of latitude 43.13°N (including mink from Niagara and Essex) as most likely to be domestic individuals. North of this latitude, the probability of being domestic depended on mink farm density. South of 43.13°N, however, latitude was the only important predictor of domestic mink probability, indicating that there is a patchy distribution of free-ranging domestic mink, independent of mink farm density and proximity. This may be due to idiosyncratic effects arising from biosecurity on particular farms. Our findings highlight what is apparently poor biosecurity on mink farms in two regions in particular, Niagara and Essex, compared to other mink farming regions in Ontario. Interestingly, Niagara and Essex were both included in the previous study by Kidd et al. (2009). The more extensive sampling in the present study has resulted in a reduction in the estimate of the prevalence of domestic and hybrid mink in Ontario (18%) compared to the previous study (39%; Kidd et al. 2009). As we increased our sampling away from southwestern Ontario, the prevalence of domestic mink in our samples decreased.

Although free-ranging domestic mink did seem restricted to areas near mink farms, our findings were consistent with previous research in showing that domestic individuals do escape from farms within their native range and subsequently interact with wild mink (Kidd et al. 2009). In particular, the processes of hybridization and introgression between domestic and wild mink are underway (Kidd et al. 2009), which will potentially have implications for the individual fitness of wild mink. Domestic mink are artificially selected for various traits, including size and pelage characteristics (Joergensen 1985; Tamlin et al. 2009), and likely exhibit relaxed selection for other traits due to captivity (Lynch and Hayden 1995). Loss of natural and sexual selection, along with genetic drift and founder effects arising from the introduction of mink to farms, can lead to a loss of local adaptation and thus reduced fitness through a variety of mechanisms and traits. To provide one anecdotal example, we studied an escaped domestic mink in Essex that foraged on low-quality foods (Dreissenid mussels) that damaged its teeth and appeared to lead to an unusually high body burden of polychlorinated biphenyls (Bowman et al. 2012). Introgression of domestic alleles into wild mink may lead to outbreeding depression (Rhymer and Simberloff 1996). We speculate that the combination of domestic mink having lost local adaptations for the wild context of their native range, and subsequent outbreeding depression following hybridization and introgression with wild mink, may explain the lack of an extensive invasive footprint of domestic mink in Ontario. Our future research plans include testing this idea using a clinal analysis of phenotypes and genotypes, where we should observe selection against domestic phenotypes for ecologically important traits (Barton and Hewitt 1985). We also suggest that further evaluation of the role of wild mink in limiting the spread of domestic mink in North America is warranted.

Hybridization and introgression are not the only processes through which domestic mink can affect wild mink populations. Escaped domestic mink could potentially compete for resources with wild mink. Although we are aware of no data demonstrating this effect in North America, feral mink have been shown to compete with the European polecat (*Mustela putorius*) and the European mink (*M. lutreola*) (Sidorovich et al. 1999; Sidorovich and Macdonald 2001). Additionally, domestic mink may play a role as a reservoir or vector for pathogens that affect wild mink (Bowman et al. 2007). This effect has been demonstrated in Ontario, where seroprevalence in mink for antibodies to Aleutian Disease Virus (ADV) is best predicted by the proximity of mink farms (Nituch et al. 2011). Furthermore, it is clear from viral sequence data that ADV spills back and forth between domestic and wild mink (Nituch et al. 2012). Although ADV seroprevalence is highest near mink farms, the virus occurs throughout our Ontario study area, and the spatial epidemiology is not fully understood. As such, it remains possible that the footprint of domestic mink in Ontario is more extensive than mink genotypes would indicate due to the role of domestic mink in transmission of ADV, and potentially other pathogens.

### Population genetics of wild mink in Ontario

There have been few studies of the population genetics of wild mink within their native range (Belliveau et al. 1999; Stevens et al. 2005). Using 245 wild genotypes from our Ontario study area, we found no isolation-by-distance, but some evidence for spatial population structure. There was weak support in our Structure analysis for seven genetic clusters, including three wild clusters: one in the northern part of the study area, one that was widespread across the central and southern part of the province, and one that was largely restricted to the Niagara peninsula. It was clear, however, that there were no absolute barriers restricting gene flow, as numerous migrants between these regions and hybrids of the wild clusters were observed. This is not surprising, as mink are vagile dispersers (Mitchell 1961), and their semiaquatic nature likely facilitates extensive movement via interconnected water bodies (Ruiz-Olmo et al. 1997; Stevens et al. 2005). The fact that the American mink is somewhat generalized in its habitat and prey requirements (Bonesi and Macdonald 2004) may also limit the extent to which population structure can develop, as it can occupy a broad and continuous range of habitat. Additional landscape genetic analyses of these wild mink genotypes may be required to reveal the underlying cause of the population structure we observed.

Although the Northern wild cluster possessed the largest number of private alleles, the Southern cluster (on the Niagara Peninsula) was the most distinct based on *F*_ST_ (Table 4). Despite the high frequency of farm escapees in this area, evidence indicates that this cluster is comprised of wild mink. One potential explanation is the Niagara River, which poses a barrier to dispersal for some terrestrial species (e.g., raccoon, *Procyon lotor*; Cullingham et al. 2009). The river may pose a moderate, but incomplete, barrier to dispersal for the semiaquatic mink, such that the Southern wild cluster is the result of dispersal across the Niagara River from a population in the northern United States. However, we detected very few individuals belonging to this cluster across the remainder of our study area. It is possible that the shape of the Niagara peninsula or the extensive agriculture in the region prevented dispersal out of the peninsula. In addition, mink that successfully cross the Niagara River may encounter a landscape saturated with escaped domestic individuals, leading to competition, disease, or outbreeding depression (Kidd et al. 2009; Nituch et al. 2011).

We observed heterozygote deficiency in several color phases of domestic mink sampled on farms. This was expected due to line-breeding practices of mink breeders (Kidd et al. 2009). We also observed heterozygote deficiency in free-ranging mink, however, from two of the sample sites (Niagara and Timiskaming). This apparent deficit of heterozygotes may be attributed to a Wahlund effect, due to overlapping populations with different ancestry. Both of these sites had wild individuals with a mixture of ancestry, and Niagara, in particular, had a large proportion of domestic and domestic–wild hybrid individuals.

Profiling of the historic mink samples from museum collections suggested that genetic diversity, as measured by expected heterozygosity and allelic richness, had not changed significantly in the wild population despite our evidence that domestic individuals are escaping and interbreeding with wild individuals. We did find, however, three unique alleles in the historic samples that were not present in any contemporary individuals. Considering that only 21 individuals were profiled, this may represent a substantial portion of the genome (i.e., the unique alleles represented 3% of all the alleles sampled in the historic mink). Although the occurrence of unique alleles in these older mink samples may be due to random genetic drift or incomplete sampling, it possibly reflects a true loss of diversity due to introgression of domestic genotypes from mink farms. Furthermore, the historic samples show that the overall genetic character of free-ranging mink in Ontario has shifted to be relatively more domesticated (mean [SE] wild membership of 0.93 [0.03] and 0.82 [0.02] for historic and contemporary free-ranging mink, respectively). Profiling of additional historic samples, particularly from regions with high densities of mink farms, may assist in elucidating the impact of mink farming on the genetic composition of contemporary wild mink populations.

## Conclusions

We found that escaped domestic mink in Ontario appear to occur mainly within the region of mink farm activity, and we found no evidence of established populations of feral mink in areas without mink farms. There was evidence, however, of hybridization and introgression between domestic and wild mink; thus, escaping domestic mink are having some impacts on wild mink at the genotypic level. These impacts appear to be most profound near mink farms, and we speculate that the spread of domestic alleles may be limited by outbreeding depression due to the maladaptation of domestic mink.

Our study also suggests that not all mink farms are equal when it comes to biosecurity. South of 43.13°N, mink farm density was not a predictor of free-ranging domestic mink; instead, there was a patchy distribution of domestic genotypes that we attribute to biosecurity differences among mink farms. In particular, we observed that two farmed areas (Niagara and Essex) appeared to be the major locations driving the presence of domestic individuals and genotypes in the free-ranging Ontario population, whereas few escapees or hybrids were detected in other high-density mink farm areas. This finding suggests that there are some mink farming regions in Ontario with successful biosecurity systems, and thus, it seems that with appropriate biosecurity in place, mink farms can mitigate the problem of escaping domestic mink.

Given that the distribution of domestic mink and their alleles is restricted to the landscape around mink farms in Ontario, and that there is no evidence of domestic mink establishing without supplementation from farms, we promote the idea of controlling invasive feral mink through biosecurity, rather than implementing an eradication program. We expect that limiting further supplementation of feral mink populations through escape or release of domestic mink should serve to eventually reduce feral mink populations in Ontario. We also promote the idea of further research on this topic in other jurisdictions in North America to assess the generality of our conclusions.
